# A Compact MIMO Antenna with Improved Isolation for ISM, Sub-6 GHz, and WLAN Application

**DOI:** 10.3390/mi13081355

**Published:** 2022-08-20

**Authors:** Batchingis Bayarzaya, Niamat Hussain, Wahaj Abbas Awan, Md. Abu Sufian, Anees Abbas, Domin Choi, Jaemin Lee, Nam Kim

**Affiliations:** 1 Department of Information and Communication Engineering, Chungbuk National University, Cheongju 28644, Korea; 2 Department of Smart Device Engineering, Sejong University, Seoul 05006, Korea

**Keywords:** MIMO antenna, isolation improvement, tri-band, compact electronics

## Abstract

This paper presents a compact two-element MIMO antenna with improved isolation for triple-band applications. The antenna consists of two radiating elements with the shared ground plane and a novel decoupling structure. Each antenna element has three stubs with different lengths, which work as quarter-wavelength monopoles to give a triple-band operation. The decoupling system is made by etching various slots in an inverted H-shape stub attached to two quarter-circles at its lower ends. The simulated and measured results show that the antenna operates (|S_11_| < −10 dB) at the key frequency bands of 2.4 GHz (2.29–2.47 GHz), 3.5 GHz (3.34–3.73 GHz), and 5.5 GHz (4.57–6.75 GHz) with a stable gain and radiation patterns. Moreover, the MIMO antenna shows good isolation characteristics. The isolation is more than 20 dB, the envelope correlation coefficient is <0.003, and diversity gain is 9.98 dB, within the frequency band of interest. Furthermore, the MIMO antenna has a compact size of 48 mm × 31 mm × 1.6 mm. These features of the proposed antenna make it a suitable candidate for I.S.M., 5G sub-6 GHz, and WLAN applications.

## 1. Introduction

Multiple input multiple output (MIMO) configurations are broadly applied to improve antenna performance, especially in terms of reliability, higher data rate, and diversity to reduce the risk of interference with other wireless devices. Instead of using wideband antennas, researchers put efforts into designing resonation at multiple bands of their own choice [1]. Along with that, more attention is put into developing a relatively small-sized antenna to meet the requirements of modern communication devices, such as cellular phones, smartwatches, portable devices, and other wearables. However, placing multiple elements of the MIMO antenna close to each other to achieve miniaturization results in a higher correlation among antenna elements [2,3]. This challenge can be mitigated using various decoupling techniques [4,5]. However, due to size constraints, these methods need further modification for efficient antenna designs.

Recently several works reported on the improvement of the isolation of the MIMO antennas [6,7,8,9,10,11,12,13,14,15,16]. For instance, the antennas reported in [6,7] present dual-band MIMO systems for 2.4 and 5 GHz applications. The multi-layered decoupling structure is utilized in [6], while a slotted plane is used to reduce mutual coupling between the consecutive elements in [7]. Although both works offer high gain along with low mutual coupling and a very low envelope correlation coefficient (ECC), the antennas have large dimensions of 160 × 160 mm^2^ and 104 × 104 mm^2^, respectively. Contrary to this, compact size dual-band MIMO antennas are reported in [8,9]. These antenna use truncated ground plane and shorted ground planes with loaded split ring resonators (S.R.R.) to reduce the mutual coupling. However, these works suffer from a high ECC value along with mutual coupling <−20 dB, which limits their applications for modern-day applications. On the other hand, a quad-band and pentaband MIMO antenna are reported in [10] and [11], respectively. These designs have the advantages of compact size and moderate gain value. They have a narrow bandwidth, high ECC and high mutual coupling [10], and high volume due to the usage of frequency selective surfaces with an air gap [11].

Various MIMO antennas for tri-band applications were proposed [12,13,14,15,16]. In [12,13] the slotted ground plane technique is exploited to achieve isolation of <18 dB, along with a very low value of ECC (<0.002) in all operational bands. However, these works have the drawback of relatively larger dimensions of 50 × 50 mm^2^ and 50 × 70 mm^2^, respectively. In addition to this, a compact antenna with a slotted ground plane is designed in [14] at the cost of low isolation. Furthermore, physical spacing between consecutive elements of the antenna system is utilized to achieve low mutual coupling, resulting in a large physical dimension. Moreover, the reported work in [15] also has setbacks of a narrow bandwidth and low gain. The MIMO antenna having a low value of ECC, and mutual coupling of >−22 dB were reported in [16]; still, it has a large size due to separated ground planes, along with low gain and narrow bandwidth.

After concluding the whole discussion, it can be observed that there is still room for further improvement of the multi-band MIMO antenna system to achieve compactness in size while keeping low mutual coupling and ECC, along with high diversity gain and broad bandwidth at all resonances. This work employs the slotted ground plane technique to improve the performance of a triple-band MIMO antenna. The antenna offers moderate gain, having a minimum isolation of 20 dB and ECC value of <0.003 in all bands.

The rest of the manuscript is organized as follows: Section 2 explains the working of the unit element of the MIMO antenna system along with the mutual coupling reduction technique. Then, various performance parameters are analyzed in Section 3. Finally, the manuscript is concluded in Section 4.

## 2. Antenna Geometry and Design Methodology

### 2.1. Geometry of the Proposed MIMO Antenna

**Figure 1:**
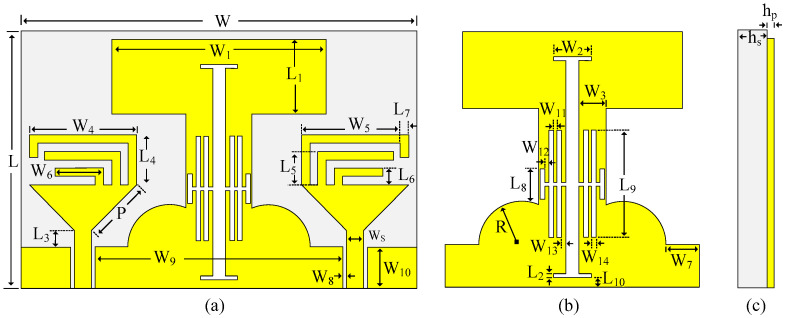
MIMO Antenna geometry: (**a**) top-view (**b**) zoomed view of decoupling structure (**c**) side view.

The geometrical configuration of the proposed low mutual coupling tri-band MIMO antenna is depicted in Figure 1. The antenna geometry is embedded on the top side of FR4, having a dielectric constant $\varepsilon_{r}$ = 4.4, the loss tangent of 0.02 with an overall size of 44 × 31 × 1.6 mm^3^. The unit element of the proposed MIMO antenna consists of a triangular-shaped quarter-wave monopole antenna fed using a co-planar waveguide (CPW) feeding mechanism, owing to the advantages of compactness and uniplanar structure [17,18,19]. Three inverted L-shaped stubs are loaded at the top side of the radiator to achieve lower band operation. Afterward, a MIMO antenna is realized by replicating the unit element. Moreover, a common ground plane is modified using slits and slots to reduce the mutual coupling between the antennas. The antenna is simulated and optimized at each step using Electromagnetic Solver HFSS, to achieve the best possible results. Table 1 shows the optimized parameters of the proposed antenna for the best performance.

### 2.2. Design Methodology

**Figure 2:**
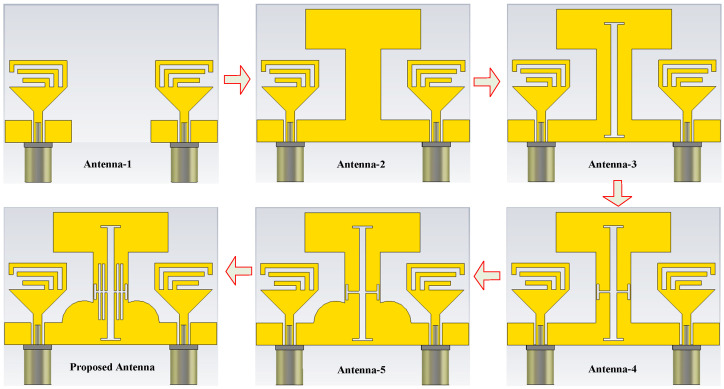
Various antennas involve in the design of the proposed tri-band MIMO antenna.

**Figure 3:**
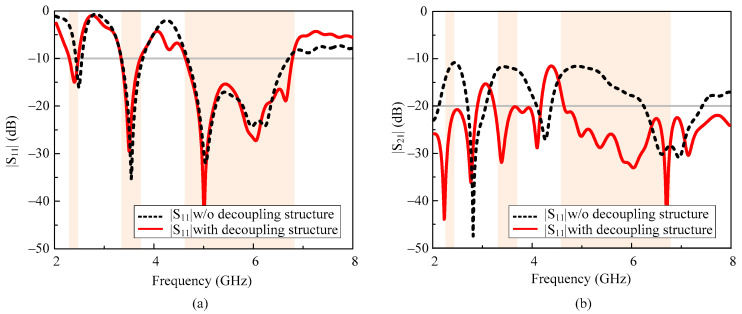
S-parameters of the proposed tri-band MIMO antenna with and without decoupling structure (**a**) |S_11_| (**b**) |S_12_|.

The geometric configuration of various antenna designs involved in the generation of the proposed MIMO antenna is depicted in Figure 2. Initially, a two-element MIMO antenna (Antenna-1) is designed by replicating the unit element in an inverted position at the distance ≈ $\frac{\lambda_{O}}{4}$, where λ*_O_* is the wavelength at the lower resonating frequency of 2.4 GHz. However, the antenna offers high coupling among the MIMO elements due to close spacing, as depicted in Figure 3. Thus, to minimize the coupling, an extended H-shape ground plane (Antenna-2) was made such that both elements share a common ground, as shown in Figure 2. This technique results in high isolation at the higher frequency bands. However, at lower resonances, the antenna offers high correlation.

**Figure 4:**
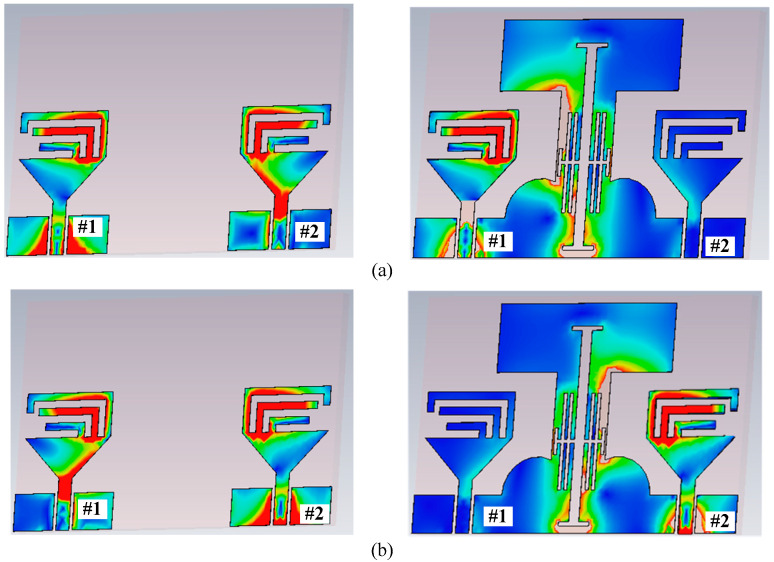
Surface current distribution among antenna with and without decoupling at 2.4 GHz: (**a**) port-1 is excited (**b**) port-2 is excited.

To improve the isolation at the lower bands, an H-shaped filtering slot is etched from the ground plane (Antenna3, 4, 5, and 6), whose effective length is adjusted by using a symmetric pair of H-shaped slots. The effect of these slots can be seen in Figure 4. It can be observed clearly that these slots converge the current distribution instead of allowing it to pass on to the nearby antenna. This minimizes the impact of the radiating antenna on the other MIMO element, results in a reduction in mutual coupling. This phenomenon can be seen in Figure 3.

## 3. Results and Discussion

### 3.1. Fabrication and Measurement Setup

**Figure 5:**
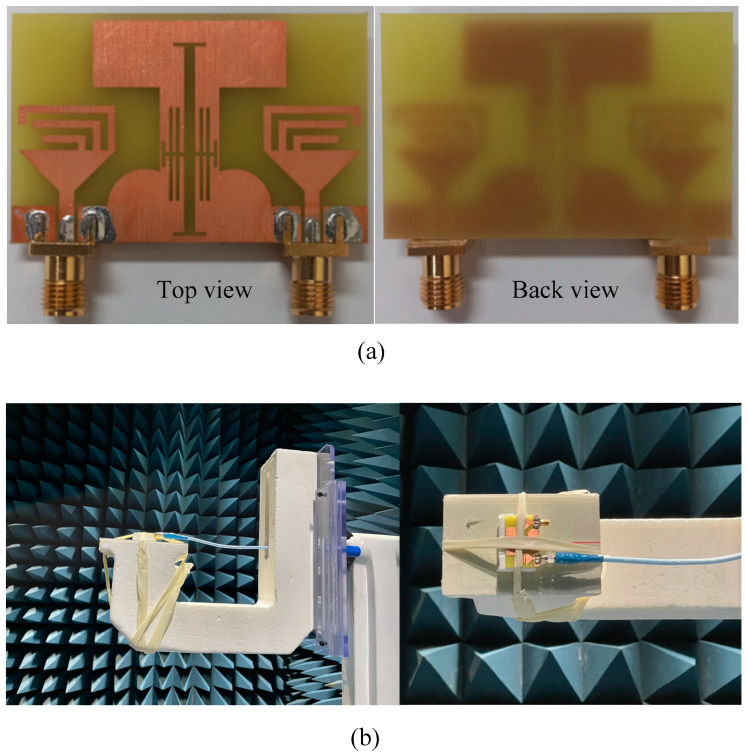
Proposed MIMO antenna: (**a**) Fabricated prototype (**b**) antenna under test.

The top and back side of the fabricated prototype of the proposed tri-band MIMO antenna is shown in Figure 5a. The antenna is fabricated using the standard chemical etching method. SMA connectors have an impedance of 50 Ω and a maximum operating frequency of 12 GHz is utilized to excite the antenna. Figure 5b shows the antenna under test in the anechoic chamber. The far-field parameters, including the radiation pattern and gain of the antenna are measured in the Anechoic chamber in Seoul, Korea [20].

### 3.2. Scattering Parameters of the Proposed MIMO Antenna

**Figure 6:**
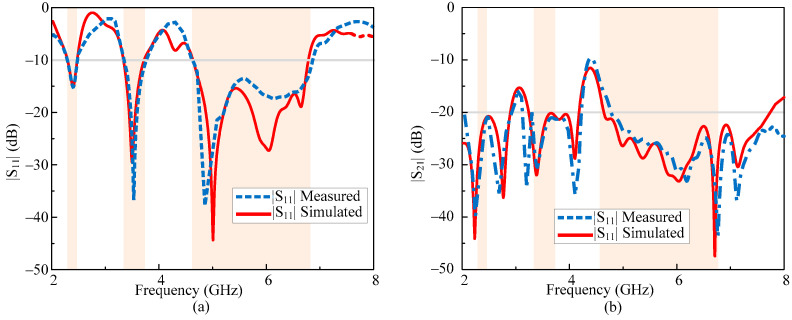
Simulated and measured S-parameters of MIMO antenna (**a**)|S_11_| and (**b**)|S_12_|.

The comparison between the simulated and measured scattering parameters of the proposed MIMO antenna is depicted in Figure 6. The antenna offers a triple-band operation ranging from 2.28–2.47, 3.34–3.73, and 4.57–6.77 GHz at the respective central frequency of 2.4, 3.5, and 5.5 GHz. On the other hand, the antenna offers a minimum isolation of 22, 20, and 23 dB while there is a maximum isolation of 36, 32, and 46 dB in respective pass bands of 2.4, 3.5, and 5 GHz. Moreover, the measured results are in a good agreement with the predicted results showing the stability of the antenna performance.

### 3.3. Far-Field Parameters

**Figure 7:**
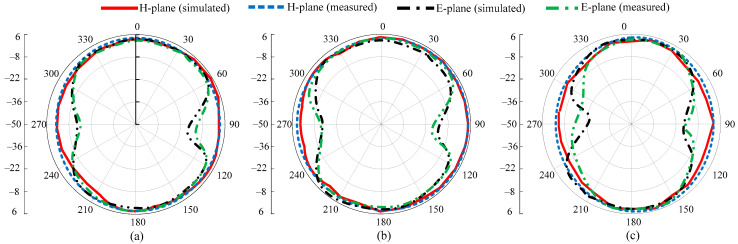
Radiation patterns of the proposed MIMO antenna: (**a**) 2.4 GHz, (**b**) 3.5 GHz, (**c**) 5.5 GHz.

The radiation pattern of the proposed MIMO antenna at the selected frequencies of 2.4, 3.5, and 5.5 GHz are shown in Figure 7a–c. The antenna offers a nearly omnidirectional radiation pattern in the principal H-plane and the dumbbell shape at the E-plane for all the selected frequencies. A little deviation is observed at a higher frequency due to an increase in the electrical length. An overall strong comparison among simulated and measured results is observed for all frequencies, as depicted in Figure 7.

**Figure 8:**
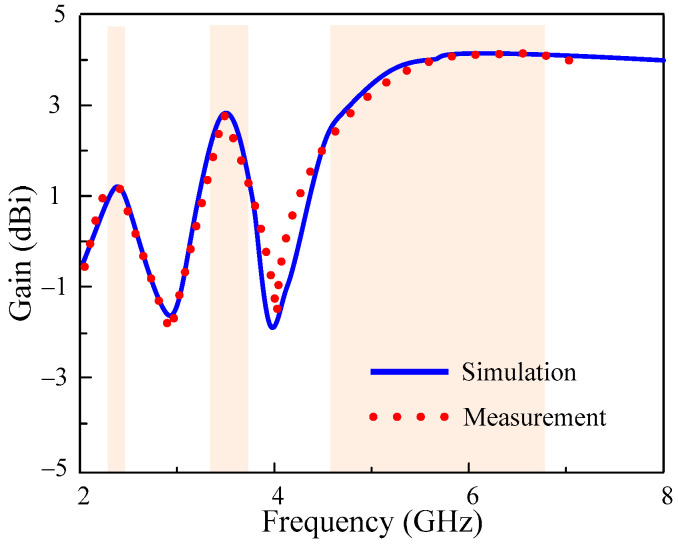
Simulated and measured gain of proposed MIMO antenna.

Figure 8 plots the simulated and measured gain of the proposed antenna. The MIMO antenna offers a peak gain of 1.3 dBi, 2.9 dBi, and 4.3 dBi, respectively, at the first, second, and third pass bands. The strong comparison among predicted and measured results shows the performance stability of this design.

### 3.4. Diversity Performance of MIMO Antenna

The study of diversity parameters becomes critically essential in MIMO antennas [21,22]. Therefore, the envelop correlation coefficient (ECC) and diversity gain (DG) of the antenna are computed. The ideal value of ECC should be zero while DG should be < 10 dB [23]. However, due to losses, the acceptable value of ECC is >0.5 and DG ≈ 10 dB in practical scenarios. For any MIMO system, the value of the ECC and DG can be calculated in terms of scattering parameters by using the following relationship provided in [24] and [25,26], respectively.

(1) $$ECC=\frac{\;{\left|S_{11}^{*}S_{12}+S_{21}^{*}S_{22}\right|}^{2}}{\left(1-{\left|S_{11}\right|}^{2}-{\left|S_{21}\right|}^{2}\right)\left(1-{\left|S_{22}\right|}^{2}-{\left|S_{12}\right|}^{2}\right)}$$

(2) $$DG=10\;\sqrt{1-{\left(ECC\right)}^{2}}\;$$

**Figure 9:**
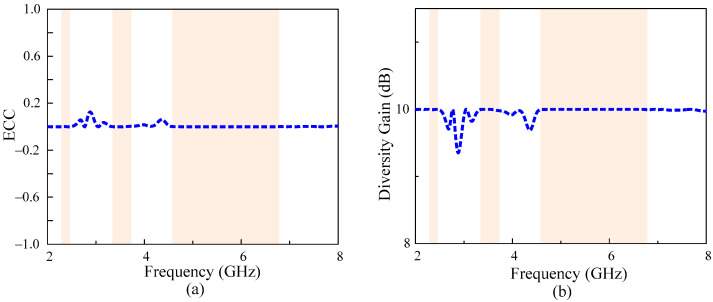
Diversity performance of the antenna: (**a**) ECC and (**b**) diversity gain.

Figure 9 shows that the antenna offers a very low value of ECC < 0.003, while a diversity gain of >9.98 dB is observed for all passbands, making the proposed work a potential candidate for diversity applications.

### 3.5. Comparison with State-of-the-ArtWork

The comparison of the proposed tri-band MIMO antenna with related work published in the literature for similar applications is summarized in Table 2. The antennas reported in [6,7,8,9] are dual-band, along with low isolation among the MIMO elements. On the other hand, the antenna reported in [10] and [11] offer quad-band and penta-bands, respectively. However, both designs suffer from narrowband, bigger dimensions, and a high value of ECC. Contrary to this, the designs in [12,13,14,15,16] offer tri-band characteristics with good diversity and gain performance. However, these antennas have large dimensions. Our design outperforms the tabulated antennas with its compact size, along with a low value of ECC, a moderate gain, and a minimum isolation of >20 dB. This makes it a potential candidate for compact-size electronics.

## 4. Conclusions

The design and realization of a compact size MIMO antenna is presented. A broadband triangular-shaped quarter-wave monopole antenna is initially designed and then loaded with three stubs of different lengths to achieve a triband behavior. Afterward, the antenna parameters are optimized to achieve desired resonating frequencies of 2.4, 3.5, and 5.5 GHz with maximum possible bandwidth. The unit element is further utilized to design a two-element MIMO system. The spacing between MIMO elements was kept at a quarter wavelength to achieve compactness in size, while the mutual coupling is reduced by introducing a novel decoupling structure. The antenna offers a tri-band operation ranging from 2.28–2.47, 3.34–3.73, and 4.57–6.75 GHz with stable gain. At the same time, the MIMO performance parameters show that the antenna offers an isolation of more than 20 dB, ECC < 0.003, and diversity gain > 9.998 dB. Moreover, the comparison with the state-of-the-art work shows that the proposed antenna overperformed the related work, by offering a good combination of size, bandwidth, and various performance parameters.

## Figures and Tables

**Table 1:** Optimized parameter of the proposed tri-band MIMO antenna.

Parameters	Dimension (mm)	Parameters	Dimension (mm)
W	48	W_8_	0.5
W_1_	26	W_9_	30
W_2_	4.5	W_10_	5
W_3_	1.5	W_11_	0.4
W_4_	13	W_12_	0.5
W_5_	9.15	W_13_	0.6
W_6_	5.9	W_14_	0.5
W_7_	4	L	31
L_1_	9	L_7_	1
L_2_	0.5	L_8_	3.5
L_3_	2	L_9_	12.6
L_4_	6	L_10_	1
L_5_	4	R	5
L_6_	2	h_s_	1.6
h_p_	0.035	Ws	2

**Table 2:** Comparison of the proposed tri-band MIMO antenna with related work published in the literature for similar applications.

Ref.	Isolation Improvement Techniques	Overall Size (mm^3^)	Operating Bands (GHz)	Peak Gain (dBi)	Min. Isolation (dB)	Max. Isolation (dB)	ECC	Diversity Gain (dBi)
[6]	Multi-layered decoupling structure	160 × 160 × 13	2.4–2.48 4.905–5.845	5.07 6.5	17 30	30 45	0.02 0.007	No Info.
[7]	Slotted ground plane	104 × 104 × 0.51	2.39–2.81 5–5.6	3.8 6.2	22 24	28 39	0.001 0.002	No Info.
[8]	Truncated ground plane	58 × 60 × 1.6	1.55–2.65 3.35–3.65	2.2 3.8	10 18	15 30	0.07 0.01	No Info.
[9]	Shorted ground with SRR.	32 × 20 × 0.8	3.3–7.75 7.9–12	3.2 4.6	20 20	27 35	0.01 0.01	9.91 9.95
[10]	Loaded stubs with ground plane	37 × 56 × 1.6	2.25–2.5 3.6–3.99 4.4–4.6 5.7–5.9	2.4 3.1 3.2 3.8	10 15 15 14	13 18 18 16	0.05 0.02 0.02 0.01	9.995 9.997 9.997 9.996
[11]	Frequency selective grid	50 × 70 × 11	2.2–2.45 2.71–2.92 3.07–3.19 3.44–3.72 5.34–5.56	8 2 4.5 3.8 0.5	30 33 33 45 23	42 52 40 50 28	0.052 0.053 0.051 0.05 0.06	9.96 9.95 9.96 9.96 9.96
[12]	Slotted ground plane	50 × 50 × 0.8	2.3–2.75 3.4–3.75 4.8–6	3.04 3.06 3.89	21 18 21	32 20 23	0.001 0.001 0.03	No Info.
[13]	Slotted ground with metallic strips	50 × 70 × 1.6	2.21–3.13 3.4–3.92 5.62–5.86	1.5 4.2 2.3	22 26 28	33 36 36	0.001 0.001 0.003	9.997 9.997 9.999
[14]	Slotted ground plane	40 × 40 × 1.6	2.4–2.74 3–3.91 4.9–5.8	No Info.	19 13 20	21 18 31	No Info.	No Info.
[15]	Physical spacing between elements	45 × 90 × 13	0.89–0.93 1.73–2.09 2.3–2.4	1.3 3.7 0.2	12.2 17.8 25	15.3 33 31	0.0005 0.0003 0.006	9.995 9.995 9.995
[16]	Separated Ground Plane	25 × 70 × 1.6	2.3–2.52 3.4–3.62 5.6–5.95	1.98 3.1 1.6	24 22 25	30 26 28	0.002 0.002 0.001	No Info.
This work	Slotted ground plane	44 × 31 × 1.6	2.28–2.47 3.34–3.73 4.57–6.75	1.3 2.9 4.3	22 20 23	36 32 46	0.003 0.002 0.003	9.998 9.999 9.998
